# Impact of Hybrid Fillers on the Properties of High Density Polyethylene Based Composites

**DOI:** 10.3390/polym14163427

**Published:** 2022-08-22

**Authors:** Basheer A. Alshammari, Asma M. Alenad, Fahad S. Al-Mubaddel, Abdullah G. Alharbi, Abdulaziz Salem Al-shehri, Hanan A. Albalwi, Fehaid M. Alsuabie, Hassan Fouad, Abdel-Hamid I. Mourad

**Affiliations:** 1 Materials Science Research Institute, King Abdulaziz City for Science and Technology (KACST), Riyadh 11442, Saudi Arabia; 2 Chemistry Department, College of Science, Jouf University, Sakaka 72388, Saudi Arabia; 3 Chemical Engineering Department, College of Engineering, King Saud University, Riyadh 11421, Saudi Arabia; 4 Fellow, King Abdullah City for Renewable and Atomic Energy (KA-CARE) Energy Research and Innovation Center, (ERIC), Riyadh 11451, Saudi Arabia; 5 Electrical Engineering Department, Faculty of Engineering, Jouf University, Sakaka 72388, Saudi Arabia; 6 Sabic Plastic Applications Development Center (SPADC), King Saud University, Riyadh 12373, Saudi Arabia; 7 Department of Chemistry, College of Science and Humanities in Al-Kharj, Prince Sattam Bin Abdulaziz University, Al-Kharj 11942, Saudi Arabia; 8 National Centre for Chemical Catalysis Technology, King Abdulaziz City for Science and Technology (KACST), Riyadh 11442, Saudi Arabia; 9 Biomedical Engineering Department, Faculty of Engineering, Helwan University, Cairo P.O. Box 11795, Egypt; 10 Mechanical and Aerospace Engineering Department, College of Engineering, United Arab Emirate University, Al Ain 15551, United Arab Emirates; 11 Mechanical Design Department, Faculty of Engineering, Mataria, Helwan University, Cairo P.O. Box 11795, Egypt

**Keywords:** high-density polyethylene, filler, polymer, composite, tensile testing, flexural strength, impact energy

## Abstract

The main objective of this work is to develop a variety of hybrid high-density polyethylene (HDPE) micro- and nanocomposites and to investigate their thermal, mechanical, and morphological characteristics as a function of number of fillers and their contents percentage. In this study, 21 formulations of the composites were prepared using fillers with different sizes including micro fillers such as talc, calcium carbonate (CaCO_3_), as well as nano-filler (fumed silica (FS)) though the melt blending technique. The morphological, mechanical, and thermal properties of the composite samples were evaluated. The morphological study revealed negligible filler agglomerates, good matrix–filler interfacial bonding in case of combined both CaCO_3_ and FS into the composites. Sequentially, improvements in tensile, flexural and Izod impact strengths as a function of fillers loading in the HDPE matrix have been reported. The maximum enhancement (%) of tensile, flexural and impact strengths were 127%, 86% and 16.6%, respectively, for composites containing 25% CaCO_3_ and 1% FS without any inclusion of talc filler; this indicates that the types/nature, size, quantity and dispersion status of fillers are playing a major role in the mechanical properties of the prepared composites more than the number of the used fillers.

## 1. Introduction

Incorporation of fillers into the polymer matrices is one of the approaches to enhance the properties and performance of resultant polymer composites. Most polymers are incompatible and immiscible in their virgin nature, but their composites exhibit several properties and can have a broad range of heterogeneous structures and morphologies. Incorporating fillers is a well-developed and established method for improving the properties of polymers, including strength, hardness, rigidity, viscosity and conductivity; it relies on not only the nature and types of polymers (thermoplastic or thermosetting) but also the shape, size, distribution, and nature of the fillers added to enhance properties [1,2,3,4].

High-density polyethylene (HDPE) is one of the most common plastics because it has some unique properties like great flexibility, good process ability and low cost; these features making it attractive for different local and industrial applications in Saudi Arabia, and it belongs to the class of polyolefin; it has an easy mold ability, low cost and density, ability to recycle and low friction during melt compounding process; however, it has relatively poor mechanical properties to be used for specific application [5,6,7,8]. Incorporating fillers into polymer matrices is industrial practice to improve polymer properties. Fillers are added to increase the bulk polymer, reduce costs, and improve the properties of the polymer. A reasonable amount of filler can improve certain mechanical or physical properties of polymers. Varieties of fillers have been recently used to fabricate reinforced polymer blends. Significant efforts have been made by the industrial and research communities to enhance the properties of polymer blends/composites. Such fillers can modify the tensile strength, hardness, rigidity, thermal stability, viscosity, and color of polymers. Many researchers have discussed an important factor in preparing polymeric composite materials, which is the proper selection of compatibilizer/coupling agents that enhance interfacial adhesion between the polymer matrices and fillers in order to improve the final properties of such composites [9,10,11,12].

Some inorganic/mineral powders commonly used as fillers in polyolefin compounds are CaCO_3_, glass powder, carbon-based powders, metal powders (copper, aluminum, and others), alumina trihydrate, talc, mica, wollastonite, silica (in all possible forms), and clays. The structural differences between both components result in the formation of large filler agglomerates in the polymer matrix, which influences the mechanical response of the materials [13,14,15,16].

Talc is the most commonly used filler for HDPE composites owing to its lamellar structure; its use often results in a reinforcing effect in polymer composites; it is composed of hydrated Mg silicate, with the chemical formula Mg_3_Si_4_O_10_(OH)_2_. Munir et al. [13] reported the enhancement of the mechanical and microstructural properties of HDPE using different fillers. The authors assert that HDPE has great process ability, enabling the easy addition of different natural and synthetic fillers. Karrad et al. [14] studied the addition of talc in HDPE composites with PP and concluded that the addition of talc enhances the properties of the polymer blends under study. Chen et al. [15] added nano-fillers in a polymer composite and discovered that nano-sized talc has significant effects on the properties of polymer composites; they demonstrated that nano-sized talc is a great filler and reinforcing agent for polymeric composites.

Calcium carbonate (CaCO_3_) is a filler that is commonly used in different blends; it mainly enhances the hardness and rigidity of polymer composites. Zebarjad et al. [16] reported that the addition of CaCO_3_ filler significantly improves the melting point, crystallinity, and heat of melting of HDPE; they obtained the most significant result using nano-fillers of CaCO_3_. Sudar et al. [17] investigated the kinetics and mechanism of the formation of voids in CaCO_3_-filled PE composites, and they found that the quasi-static mechanical properties were improved.

According to recent studies, the mechanical properties of various thermoplastic-based polymeric composites can be enhanced using FS without compromising the optical properties of the composite. For instance, Zhang [18] investigated the effect of nano-silica on the mechanical properties of HDPE, and they found that it increases the tensile strength, impact strength, and tensile stiffness, confirming that adding fillers can tailor the properties of polymeric composites. Kontou et al. [19] added nano-FS particles in LDPE, and the mechanical properties of the composite were improved compared to those of the polymer. Dorigato et al. [20,21] investigated the effect of silica fillers on the properties of HDPE, especially the thermo-mechanical properties, and they recorded a more prominent effect in the polymer matrix with a high surface area of filler. The obtained silica-filled composite exhibited good thermal and mechanical properties and had good dimensional stability [22].

Several researchers have prepared HDPE composites with different number of the fillers and diverse processing techniques in order to improve the properties and reduce the manufacturing cost of polymer composites products. Further, the effect of single or two fillers on the characteristics of HDPE has been studied enormously. For example, effect of blending technique of zinc oxide (ZnO) nanoparticles with HDPE on HDPE/ZnO nanocomposites has been investigated by Benabid et al. [23]. Chen et al. [15], Zebarjad et al. [16] and Kontou et al. [19] investigated Talc, CaCO_3_ and FS as single fillers into HDPE, respectively. The results of these study show that single filler has a positive effect on both thermal and mechanical properties of HDPE composite materials and each filler has a specific feature to contribute to improvements of such properties. Some other works have also been conducted for investigating the impact of the inclusion of two fillers on the properties and performance of the HDPE polymer matrix. For instance, Guo et al. [24] have studied the significant effect of wood fiber/carbon fiber hybrid fillers on the mechanical and physical characteristics of HDPE-based composites. Morphology, mechanical and thermal performance of HDPE reinforced with hybrid inorganic Huang et al. [25] have investigated fillers (fiberglass and talk). Their findings illustrated that, the use of fiberglass has considerable impact in enhancing the mechanical properties of the composites. While the use of talc reduced the cost of the composite and improved its recyclability. Based on the above it is obvious that using single or double fillers, each filler has a specific role in enhancing the characteristics and performance of HDPE composites. The research concerned with utilizing three different fillers is very lacking and need to be investigated.

The aim of current work is to study the impact of three different fillers both talc and CaCO_3_ as micro-fillers as well as a fumed silica (FS) as nano-filler on the mechanical, chemical and physical characteristics of HDPE polymer matrix to fabricate different formulations of HDPE-based composite materials; this study focused on improving the tensile, flexural, and impact strengths for developing a material composite that could be used in specific industrial applications such as building and construction as well as wind turbine blades as commonly used polymers, including polyolefin, by adding such inorganic fillers into these polymers, something which has great potential in different industrial applications.

## 2. Materials and Methods

### 2.1. Materials

Twenty-one HDPE composite samples were prepared. The compositions of the prepared composites are listed in Table 1. The HDPE was supplied by the Saudi Arabian Basic Industries Corporation (SABIC), Riyadh, Saudi Arabia; it has a melt flow index (MFI) of 8 g/10 min (MFI @ 2.16 kg & 190 °C) and density of 964 kg/m^3^. Cyclic olefin copolymer Extend the abbreviation (COC) was supplied also by SABIC. In order to enhance stiffness and moisture resistance [26], 20% of COC was blended with HDPE polymer for all samples investigated and the blend named as HDPE in this study. Calcium carbonate (CaCO_3_) powder grade Omega (average particle size 5 μm), supplied by Lime Quality Co., Ltd. (Bangkok, Thailand). The talc filler (Mean particle diameter = 6.3 μm) was supplied by Chung Chemicals Sdn. Bhd (Kuala Lumpur, Malaysia). Evonik specialty chemicals supplied fumed Silica (FS) grade (AEROSIL 200, with an average particle size of 12 nm).

### 2.2. Composite Preparation Techniques

The HDPE resin/matrix and fillers were dry-blended using a 5 Kg capacity laboratory mixer (Henschel, Kassel, Germany) to homogenize the formulations. The mixing time was 5 min, and the rotor speed was 60 rpm. After dry blending, the blends were melted and formed using a compression-molding machine in order to obtain sheets having 4 mm thickness. The specimens for all tests were cut from the compression molded samples using a contour cutter (Model Ray-Ran, Hertfordshire, United Kingdom).

### 2.3. Formulations of the Composite

Employing the melt blending technique, the 21 formulations of HDPE/filler composites were prepared using the three fillers (talc, CaCO_3_, and FS) as shown in Table 1. The formulations divided to 5 groups; each group has the same loadings of nano-filler i.e., FS; however, amount of both micro filler i.e., talc and CaCO_3_ ranging from 0 to 25% with the total of both fillers 25%.

## 3. Experimental

### 3.1. Thermal Analysis

A TA Instrument Differential scanning calorimetry (DSC, Shimadzu DSC-Q100, Japan) was used to examine the melting temperature of the composites. The samples were hermetically sealed in aluminum pans, and an empty pan was sealed and used as a reference. The sealed pans were scanned from 25 °C to 150 °C in a nitrogen atmosphere using a heat-cool-heat run, at heating and cooling rates of 10 °C/min. The DSC Heat/Cool/Heat run are designed to erase preceding thermal history by heating the material above its transition (e.g., glass transition or melting), where relaxation or molecular rearrangement can occur, then cooling at a known rate before heating again. To erase their thermal history, samples were kept at 150 °C for 5 min and then cooled down to 25 °C. From the other side, Fenni et al. [27] have used equivalent temperature and time to erase the thermal history of HDPE. Generally, in polymer science, heat history is erasable by heating the polymeric material slightly above the melting temperature. Data obtained from all runs were used for analysis. To confirm the results, three samples from each material were measured.

Furthermore, Melt Flow Index measurements (MFI) were conducted to obtain information about the rheological properties of the composites. The melt MFI was measured by a melt flow indexer based on the standard ISO 1133 with a load of 2.16 kg. The bulk density of the composite materials is determined according to ASTM D792-13 by using Archimedes’ principle. About 50 g of each composite specimen cut and immersed into the cylinder containing the distilled water. Afterward displacement of water volume is observed, and the density values of the composites materials is the calculated using the Formula (1) as following:

(1) $$\rho \;(g/{\mathrm{cm}}^{3})\;=\;\frac{m}{v}$$

where *m* = mass, *ρ* = density and *v* = volume.

Nevertheless, the MFI is not a direct measure for the viscosity, but it is a qualitative indication for which composite has a higher value or lower value of the viscosity. In other wording, it has a correlation with viscosity. Furthermore, melt shear viscosities were measured using a TA Instruments rheometer (model AR 2000). A strain sweep test was primarily conducted to define the linear viscoelastic region of the composite materials. The dynamic frequency sweep test (strain: 0.3%; frequency: 0.1 to 1000 rad/s; and room temperature at shear rates ranging from 50 to 1000 s^−1^).

### 3.2. Mechanical Analysis

Tensile tests were performed according to the ASTM D638 specification using a testing machine (Zwick Roell, Ulm-Germany) with a load-cell capacity of 10 kN at a cross-head speed of 50 mm/min for five specimens having dimension of (120 × 10 × 1) mm and the gage length of 50 mm. The Izod impact test (notched) was performed at room temperature for the five replicate specimens of each composite samples taking the dimensions of (70 × 10 × 1) mm according to the ASTM D256 standard using a Zwick HIT50B impact testing machine (Zwick GmbH & Co. KG, Ulm, Germany). The Flexural tests were performed on Three-point bending flexural tests according to ASTM D790 standard with the crosshead speed of 2.0 mm/min were applied on upper and lower surface of each six replicate specimens of pure HDPE and its hybrid composites. The failure was calculated when bending of specimens having dimension of (120 × 10 × 1) mm and the gauge length of 50 mm reach up to corresponding critical point.

### 3.3. Scanning Electron Microscopy (SEM)

The samples were prepared by freeze fracturing the pellets in liquid nitrogen, and they were attached to 12.5-mm diameter Al stubs having sticky 12-mm diameter C tabs. The samples were coated with Au–Pd for 2 min at a deposition current of 40 mA under Ar pressure of 0.1 mbar. The samples were transferred to the SEM chamber and examined using a field electron and ion (FEI Quanta 200 (SEM-2, Oregon, USA) microscope at an accelerating voltage of 20.0 kV. In the secondary electron imaging (SEI) mode, the SEM micrographs of the samples were recorded at an image resolution of 1024 × 784, spot size 3.0, and working distance of 14–16 mm.

## 4. Results and Discussion

### 4.1. Thermal Properties

#### 4.1.1. Melting Temperature

**Figure 1:**
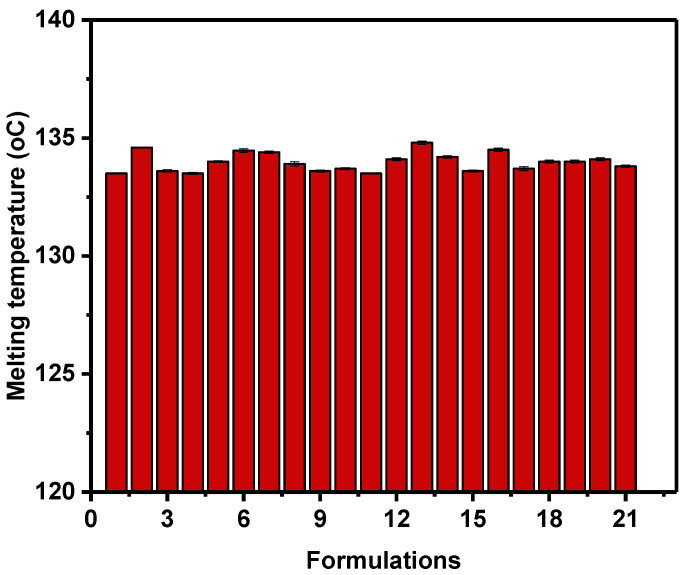
DSC-derived melting temperature (T_m_) of HDPE/filler composites.

The DSC-derived thermal degradation, representing the melting temperature (T_m_) of the prepared unfilled and filler-loaded HDPE composites, is shown in Figure 1. Formulations 2 (75% HDPE + 25% talc), 6 (75% HDPE + 25% CaCO_3_), 13 (70% HDPE + 12.5% talc + 12.5% CaCO_3_ + 5% FS) and 16 (72% HDPE + 20% talc + 5% CaCO3 + 3% FS) showed high T_m_, approximately 0.96% (1.29 °C) and 1.08% (1.44 °C) increase, respectively, compared to that of the unfilled HDPE; this implies that 12.5% talc and 12.5% CaCO_3_ in the presence of FS at the highest loading level (5%) enhances the stability of the pure HDPE matrix and subsequently improve the melting temperature of the composites materials; moreover, other formulations exhibited a similar behavior compared to that of the unfilled HDPE; this indicate that the higher amount of both talc and CaCO_3_ hybrid filler in the presence of highest amount of FS can affect the melting temperature of polymer composites not significantly; these results indicate that filler type and its loading can affect the melting temperature of the final composites materials very slightly [28]. In addition, in the work of Mourad and his research group [29,30,31,32] and in reference [33] a melting temperature of HDPE and its composites in the range 127.8–132 °C has been obtained; this range is coincident to the temperature range in Figure 1.

#### 4.1.2. Viscosity, Flow Index and Density

**Figure 2:**
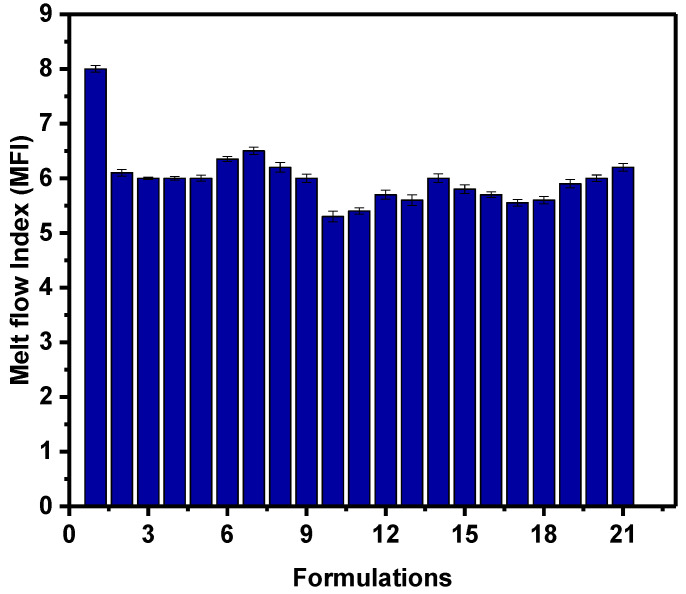
Melt flow index (MFI) of HDPE/filler composites.

**Figure 3:**
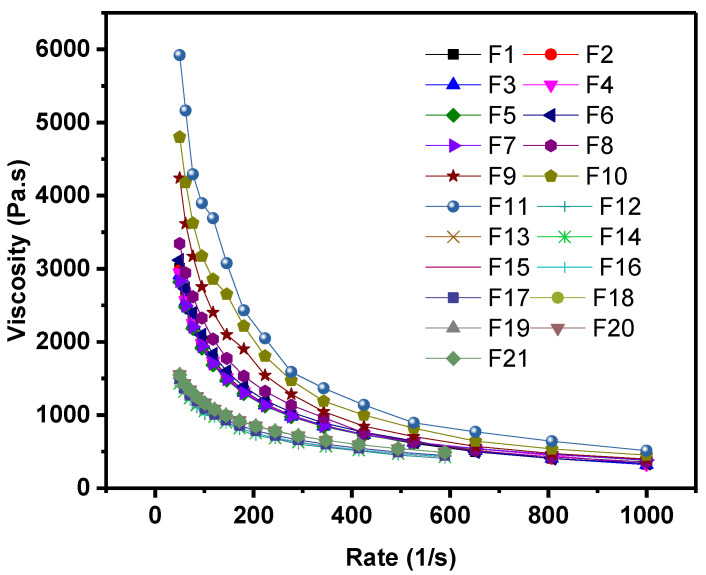
Viscosity of HDPE/filler composites.

Furthermore, MFIs of the various formulations are shown in Figure 2. Compared to pure HDPE, MFI of the composites decreased with the addition of fillers. Formulation 17 (70% HDPE + 20% talc + 5% CaCO_3_ + 5% FS) exhibiting the highest decline in MFI (~33.75); this indicates that the formulation 17 has the highest melt viscosity value compared to others. On the contest, MFI of Formulation 7 (CaCO_3_ and FS loaded at the highest (25%) and lowest (1%) concentrations, respectively, was least affected. Generally, the addition of fillers led to a decrease in the MFI. In particularly talc filler since no talc in the formulations 6, 7 and 8 which have very similar MFI values; these formulations have of 25% CaCO_3_ (group 2, Table 1) and various loading of FS (0, 1 and 3%). The reduction in MFI values is because the incorporation of fillers hinders blend flow and increases the viscosity of composites at the melt state; this indicates that talc is can be decrease MFI more than others fillers. For instance, Formulations 10 and 11 (group 3) showed the lowest MFI (Figure 2) and the highest melt viscosity (Figure 3) among formulations as they have 12.5% talc; this indicated that these formulations might demonstrate difficulty in fabrication composites compared with others, and could be attribute to the possible agglomeration of the fillers particles in the polymer matrix [34]. In the other hand, the formulation 7 showed a comparable MFI with neat HDPE (Figure 2) or lower melt viscosity compared to the formulation 17 (Figure 3). Generally, lower viscosity materials are preferable foe easy formation. On the other hand, the bulk density values for each combination of the final composites are higher than density value of HDPE matrix slightly; this may be linked to be the fact that the density of the fillers is higher than that of the HDPE polymer.

### 4.2. Mechanical Properties

#### 4.2.1. Tensile Behavior

**Figure 4:**
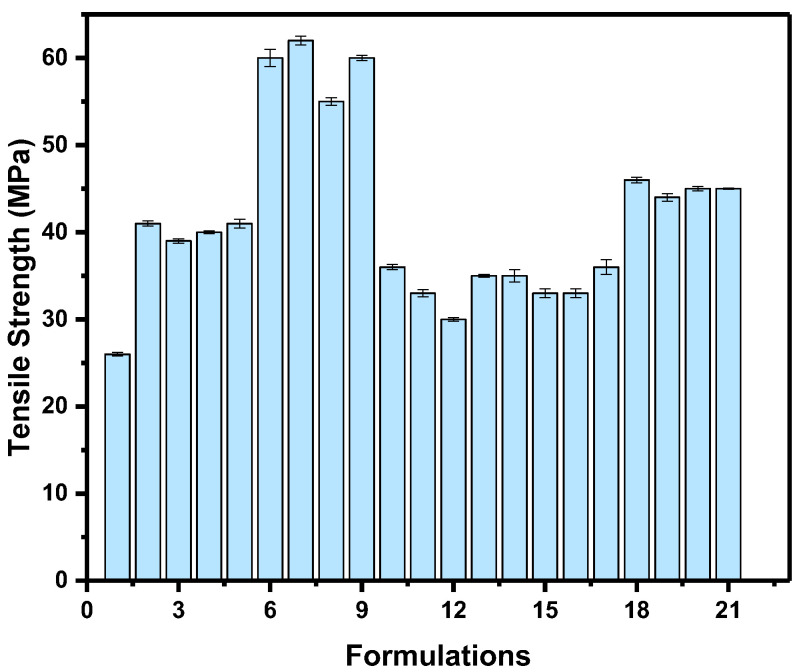
Tensile strength (MPa) of the HDPE/filler composites.

The tensile strengths of the unfilled and filler-loaded HDPE composites are listed in Table 2 and Figure 4. The tensile yield strength increased with the addition of fillers in the HDPE polymer matrix. Formulation 7 (74% HDPE + 25% CaCO_3_ + 1% FS) exhibited a maximum tensile strength (~62 MPa), of ~58% increase compared to that of unfilled HDPE. In addition, Formulations 6 (75% HDPE + 25% CaCO_3_) and 9 (70% HDPE + 25% CaCO_3_ + 5% FS) exhibited a comparable tensile strength of ~58 MPa. On contrast, Formulations 13 (70% HDPE + 12.5% talc + 12.5% CaCO_3_ + 5% FS), 15 (74% HDPE + 20% talc + 5% CaCO_3_ + 1% FS) and 16 (72% HDPE + 20% talc + 5% CaCO_3_ + 3% FS) showed the lowest tensile strength (~33 MPa), which is a ~18.75% decrease compared to that of the unfilled HDPE, as shown in Table 2; these results indicate that the addition of CaCO_3_ at high concentrations (25%) without addition of talc to HDPE polymer matrix greatly improves the mechanical properties. Mehrjerdi et al. [35] reported that, there is no effect on the composites strength for different filler concentrations include carbon black and talc. The addition of talc into the HDPE was up to 35% without any significant effect on the tensile properties of the composites; these result in a good agreement with that obtained results in this study as highest tensile strength achieved for composite materials containing only CaCO_3_ and FS without any talc (Formulation 7). The obtained tensile strength for this formulation is higher than the tensile strength (~27.5%) reported by Awan et al. [36] for CaCO_3_ reinforced HDPE matrix composite; these results agree well with the results of this work as CaCO_3_ significantly enhanced the tensile strength of the final composite materials and it is worth noting that all developed composites achieved higher tensile strength that than pure HDPE. Conversely, Khalaf et al. [8] has reported that decreasing trend for the tensile strength of CaCO_3_ reinforced HDPE composites; they attributed this reduction to the stress concentrations that started some cracks and poor dispersions and distribution, as they did not use hybrid fillers, treatment and/or modification.

The yield strength is the most important property; it is also a reliable value and can be obtained accurately with high degree of reproducibility from the tensile stress strain curves. The inclusion of the fillers affects the repeatability to some extent of the values of stiffness and elongation at break; however, the modulus of elasticity of the tested materials was found to be in the range 350–430 MPa. The composite materials have higher values than pure HDPE. The percent elongation has been measured to be in the range of 500–645% and their values reduce with filler contents. Similar results and behavior have been reported in [8,29,30,31,32,33]; this behavior for the composite materials is a result of the improved stiffness of the composites that was attributed to interaction between the HDPE and hybrid fillers and the fillers have fewer elongation values; this reduction in elongation (%) reduced the ductility, and limited the stretching of the composites. In another word, the fillers loading have restrained the HDPE polymer chains movement and resulting in the presence of highly localized strain, which cause de-wetting between the HDPE matrix and fillers. Thus, the HDPE matrix becomes stiffer and less ductile. As a result, the toughness and resilience of the final composite decreased and lead to lower elongation at break; it has been reported that the elasticity modulus, yield strength, tensile strength and hardness of HDPE composites enhanced with decreasing filler particle size and increasing its loading [13].

#### 4.2.2. Izod Impact Strength

**Figure 5:**
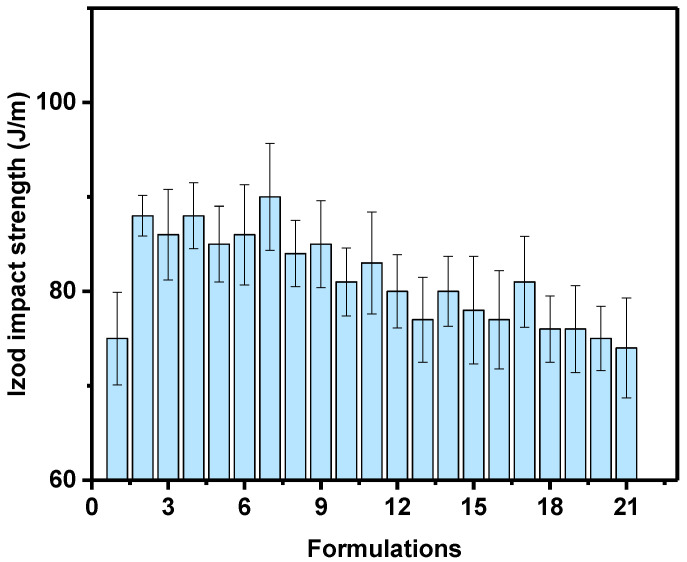
Izod impact strength (J/m) of the HDPE/filler composites.

The notched Izod impact strength/toughness results of the unfilled and filler-loaded HDPE composites are summarized in Table 2. The neat HDPE achieved an impact toughness of 75 ± 9.40 J/m. Table 2 and Figure 5 show that few composites (formulations 18–21) achieved impact toughness from 70 up to 72 that is less than that for neat HDPE (75 ± 9.40); this could be attributed to the insufficient filler within the polymer matrix; however, the additive did not affect the impact strength significantly as tensile and flexural strength. On the other hand, the sample with talc, CaCO_3_ and FS loaded at a concentration of 0, 25 and 1% FS respectively which exhibited the highest impact energy, which implies improved toughness. The obtained results are in agreement with results reported by Khalaf et al. [8] and Leong et al. [37]; they used talc and CaCO_3_ to reinforced polypropylene (PP) and HDPE composites; respectively to compare their mechanical properties; they concluded that talc tends to agglomerate at fillers loadings higher than 1%, and this causes the strength and toughness of filled PP and HDPE composite to decrease their substantially.

#### 4.2.3. Flexural Strength

**Figure 6:**
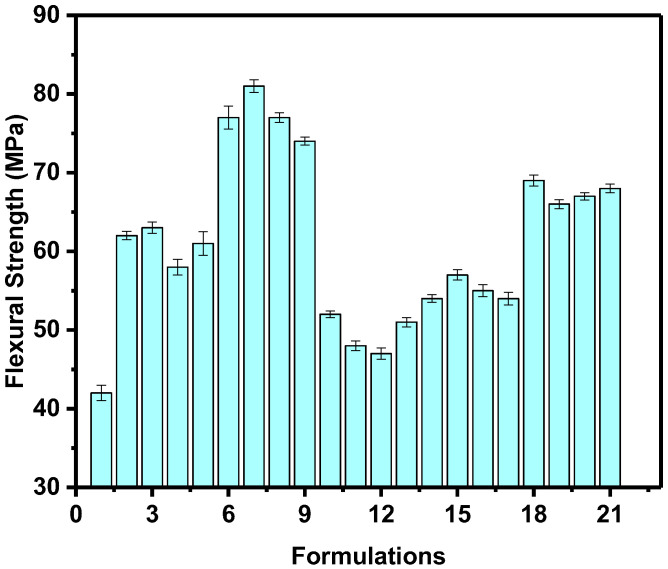
Flexural strength of the HDPE/filler composites.

The flexural behavior of the unfilled and fillers-loaded HDPE composites is presented in Table 2. The flexural strength of unfilled HDPE is 42 ± 0.96 MPa and the composites have achieved flexural higher than neat HDPE ranged from 47 ± 0.72 to 8 ± 0.81 MPa; it has been observed that an increment in flexural strength, which can be attributed to the appreciable filler–matrix interaction, which possibly enhances the transmission of stress from the matrix to the fillers, thus improving the stiffness of the HDPE/filler composites [25]. As shown in Figure 6, the maximum flexural strength was 81 MPa, which was recorded by the formulation 7; this indicates that the addition of CaCO_3_ and FS improves bonding with the polymer matrix, which favors the flexural properties of the HDPE composites. The control sample recorded the minimum flexural strength of about 42 MPa. The improvement in the mechanical properties indicate that the formulation 7 has lower chains flexibility, good dispersion and distributions of fillers into HDPE matrix, compared to other formulations. In general, there are three composites groups, group 1 (formulations 2–9) that achieved the highest values and the second group (formulations 18–21) that achieved the intermediate values. Group 3 composites have flexural strength between group 1 and 2; it is worth noting that all filled HDPE composites have strength more than neat HDPE composite.

**Figure 7:**
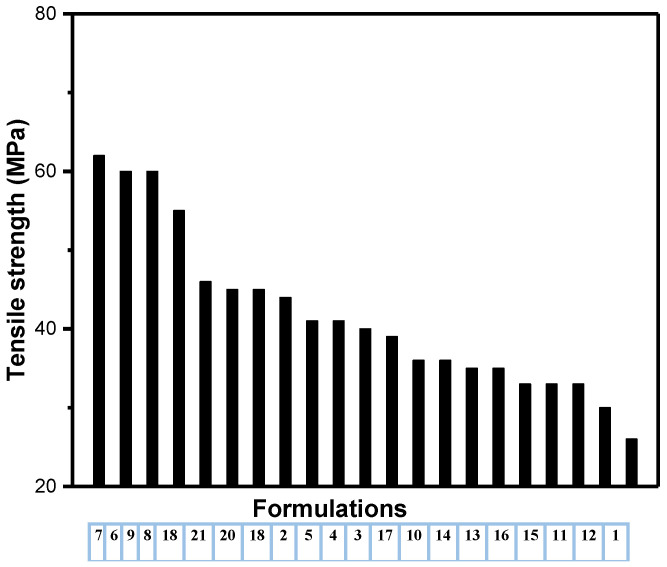
Tensile strength versus formulations in a descending order.

**Figure 8:**
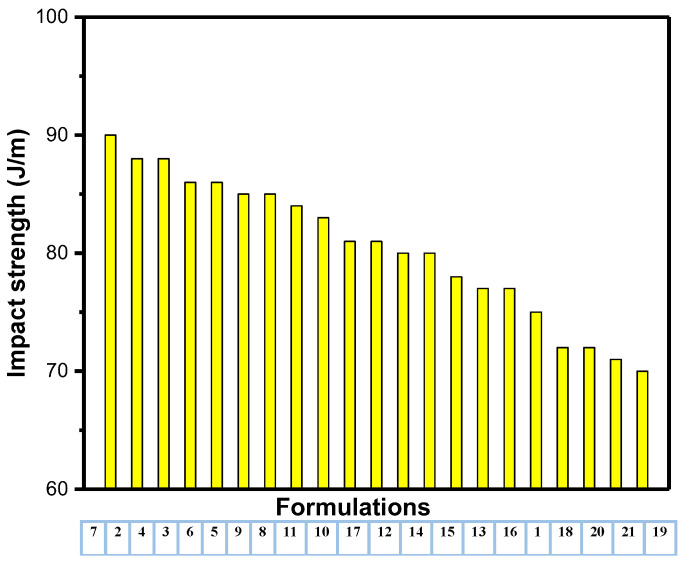
Impact strength versus formulations in a descending order.

**Figure 9:**
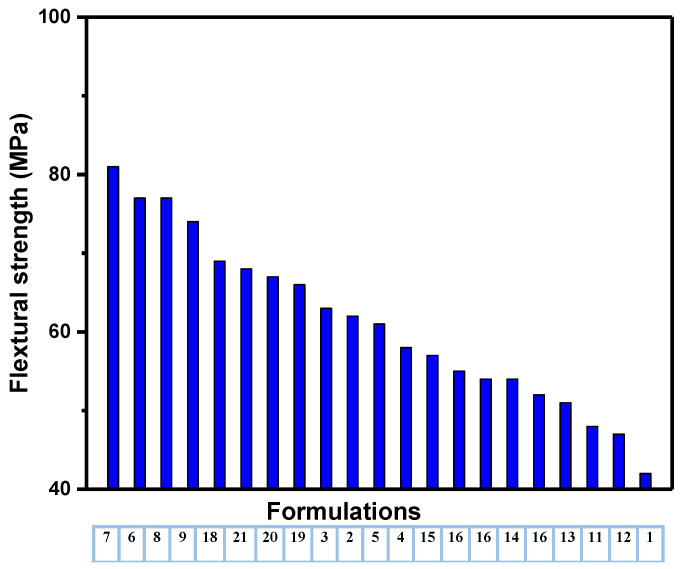
Flexural strength versus formulations in descending.

Figure 7, Figure 8 and Figure 9 show the descending values of all mechanical properties versus their formulations. From Figure 7, it is clear that composites of formulation 7 has the highest mechanical properties among others. In addition, most composites groups have similar flexural and tensile strength (formulations 1, 6–7, 11–12 and 18–21); this indicates that number of hybrid fillers into the polymer matrix may not contribute to the mechanical properties as other factors have a significant role include size, amount, shape, type, aspect ratio as well as their dispersion and distribution within the composites.

### 4.3. Morphological Properties

**Figure 10:**
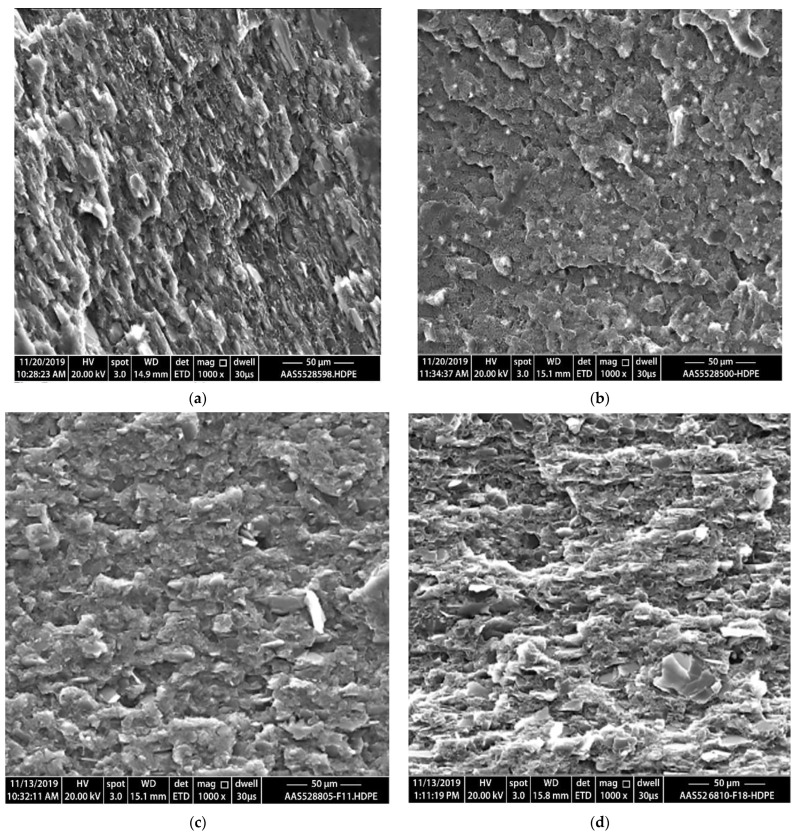
SEM images of the fractured composites (Scale bar is 50 μm for all images). (**a**) Formulation 2; (**b**) Formulation 7; (**c**) Formulation 11; (**d**) Formulation 16. The images in (**a**,**c**,**d**) indicate some clear agglomerations, voids as well as poor dispersion and distribution. While The image in (**b**) exhibit good interfacial adhesion between the fillers and the HDPE polymer matrixcompared to other HDPE/filler composites.

The SEM micrographs of the prepared composites are shown in Figure 10a–d. The SEM micrographs demonstrate some of talc filler agglomerates. For example, formulations 2, 11 and 16 in Figure 10a,c,d; respectively, show agglomeration of talc filler and poor distribution within the composites; however, formulation 7 (Figure 10b) shows better dispersion and distribution of the fillers compared with other formulations. In addition, the composite materials in formulation 7 exhibits good interfacial adhesion between the filler and the HDPE polymer matrix compared to other HDPE/filler composites; this indicates a good dispersion of the fillers (CaCO_3_, and FS) within the HDPE matrix compared with the talc filler, and leads to enhancing the interfacial filler–matrix bonding and consequently improved mechanical properties as shown for formulation 7 that achieved the highest mechanical properties compared to all other formulation.

## 5. Conclusions

In this study, different formulations of HDPE/filler composites were fabricated by melt compounding technique using three fillers (talc, CaCO_3_, and FS) with varying concentrations. The thermal, mechanical, and morphological characteristics were investigated. The MFI was observed to decreased with the addition of filler either individual or hybrid. Morphological analyses of the fracture surfaces showed good filler dispersion in the HDPE matrix, in particularly for composites having highest amount of CaCO_3_ and lowest FS with zero amount of talc, resulting in significant improvement in both the tensile, impact and flexural strengths. The maximum measured tensile, flexural and impact strengths were 62 MPa, 90 MPa and 81 MPa, respectively for composites containing 74% of HDPE, 25% of CaCO_3_ and 1% of FS please check these values; these results indicate that, the filler types and their desperation and distribution, have a significant effect on the mechanical properties of the composites rather than their numbers. The tailoring of the thermal, mechanical, and morphological properties of such composite materials could open up new avenues for producing functional high-performance polymer composites for several applications such as building and construction as well as wind turbine blades materials.

## Figures and Tables

**Table 1:** Compositions of the different HDPE/filler composites formulations.

Groups	Formulations	HDPE %	Talc %	CaCO_3_ %	FS %
Control	1	100	0	0	0
Group 1	2	75	25	0	0
	3	74	25	0	1
	4	72	25	0	3
	5	70	25	0	5
Group 2	6	75	0	25	0
	7	74	0	25	1
	8	72	0	25	3
	9	70	0	25	5
Group 3	10	75	12.5	12.5	0
	11	74	12.5	12.5	1
	12	72	12.5	12.5	3
	13	70	12.5	12.5	5
Group 4	14	75	20	5	0
	15	74	20	5	1
	16	72	20	5	3
	17	70	20	5	5
Group 5	18	75	5	20	0
	19	74	5	20	1
	20	72	5	20	3
	21	70	5	20	5

**Table 2:** Mechanical properties of pure and filled HDPE composites.

Formulation	Melt Flow Index (2.16 kg)	Density (g/cc)	Tensile Yield Strength (MPa)	Izod Impact Strength (J/m^2^)	Flexural Strength (MPa)
1	8.0	0.96	26 ± 0.20	75 ± 9.40	42 ± 0.96
2	6.1	1.14	41 ± 0.30	88 ± 2.15	62 ± 0.52
3	6.0	1.14	39 ± 0.26	86 ± 4.86	63 ± 0.71
4	6.0	1.15	40 ± 0.15	88 ± 3.49	58 ± 0.98
5	6.0	1.15	41 ± 0.51	85 ± 4.02	61 ± 1.49
6	6.35	1.13	60 ± 0.98	86 ± 5.31	77 ± 1.45
7	6.50	1.13	62 ± 0.21	90 ± 5.67	81 ± 0.81
8	6.20	1.14	55 ± 0.43	84 ± 3.5	77 ± 0.62
9	6.00	1.15	60 ± 0.28	85 ± 4.60	74 ± 0.51
10	5.8	1.14	36 ± 0.30	81 ± 3.6	52 ± 0.41
11	5.6	1.14	32 ± 0.40	83 ± 5.4	48 ± 0.62
12	5.7	1.14	30 ± 0.20	80 ± 3.9	47 ± 0.72
13	5.6	1.16	33 ± 0.15	77 ± 4.5	51 ± 0.58
14	6.0	1.15	35 ± 0.70	80 ± 3.7	54 ± 0.49
15	5.8	1.16	33 ± 0.51	78 ± 5.7	57 ± 0.65
16	5.7	1.15	33 ± 0.50	77 ± 5.2	55 ± 0.75
17	5.3	1.40	36 ± 0.85	81 ± 4.8	54 ± 0.80
18	5.5	1.20	46 ± 0.32	72 ± 3.5	69 ± 0.69
19	5.9	1.11	44 ± 0.45	70 ± 4.6	66 ± 0.59
20	6.0	1.23	45 ± 0.23	72 ± 3.4	67 ± 0.46
21	6.2	1.34	45 ± 0.51	71 ± 5.3	68 ± 0.53

## Data Availability

Not applicable.

## References

[1] Islam E., Kumar A., Hakkim N.L., Nebhani L. (2021). Silica Reinforced Polymer Composites: Properties, Characterization and Applications. Encycl. Mater. Plast. Polym..

[2] Eterigho-Ikelegbe O., Yoro K.O., Bada S. (2021). Coal as a Filler in Polymer Composites: A Review. Resour. Conserv. Recycl..

[3] Jotiram G.A., Palai B.K., Bhattacharya S., Aravinth S., Gnanakumar G., Subbiah R., Chandrakasu M. (2022). Investigating mechanical strength of a natural fibre polymer composite using SiO_2_ nano-filler. Mater. Today Proc..

[4] Vinay S.S., Sanjay M.R., Siengchin S., Venkatesh C.V. (2021). Basalt fiber reinforced polymer composites filled with nano fillers: A short review. Mater. Today Proc..

[5] Hsissou R., Seghiri R., Benzekri Z., Hilali M., Rafik M., Elharfi A. (2021). Polymer composite materials: A comprehensive review. Compos. Struct..

[6] Huang R., Zhang Y., Xu X., Zhou D., Wu Q. (2012). Effect of hybrid mineral and bamboo fillers on thermal expansion behavior of bamboo fiber and recycled polypropylene-polyethylene composites. BioResources.

[7] Slouf M., Radonjic G., Hlavata D., Sikora A. (2006). Compatibilized iPP/aPS blends the effect of the viscosity ratio of the components on the blend’s morphology. J. Appl. Polym. Sci..

[8] Khalaf M.N. (2015). Mechanical properties of filled high density polyethylene. J. Saudi Chem. Soc..

[9] Wu H.F., Dwight D.W., Huff N.T. (1997). Effects of silane coupling agents on the interphase and performance of glass-fiber-reinforced polymer composites. Compos. Sci. Technol..

[10] Lu J.Z., Wu Q., McNabb H.S. (2000). Chemical coupling in wood fiber and polymer composites: A review of coupling agents and treatments. Wood Fiber Sci..

[11] Shirin S., Arefazar A., Khosrokhavar R. (2008). Silane coupling agents in polymer-based reinforced composites: A review. J. Reinf. Plast. Compos..

[12] Pimbert S. (2003). Evaluation of the fractionated crystallization of isotactic polypropylene and high density polyethylenes in their blends with Cycloolefin copolymers. Macromol. Symp..

[13] Tasdemir M. (2012). Effects of Particle Size on Mechanical, Thermal and Morphological Properties of Untreated Nano and Micro Calcium Carbonate Powder [CaCO_3_] filled HDPE Polymer Composites. J. Polym. Mater..

[14] Kaeead S., Cuesta J.-M.L., Crespy A. (1998). Influence of a Fine Talc on The Properties of Composites With High-Density Polyethylene And Polyethylene Polystyrene Blends. J. Mater. Sci..

[15] Chen N., Ma L., Zhang T. (2006). Investigation of nano-talc as a filling material and a reinforcing agent in high density polyethylene (HDPE). Rare Met..

[16] Zebarjad S.M., Sajjadi S.A. (2008). On the strain rate sensitivity of HDPE/CaCO3 nanocomposites. Mater. Sci. Eng. A.

[17] Sudar A., Moczo J., Voros G., Pukanszky B. (2007). The mechanism and kinetics of void formation and growth in particulate filled PE composites. Express Polym. Lett..

[18] Zhang M.Q., Rong M.Z., Zhang H.B., Friedrich K. (2003). Mechanical properties of low nano-silica filled high density polyethylene composites. Polym. Eng. Sci..

[19] Kontou E., Niaounakis M. (2006). Thermo-mechanical properties of LLDPE/SiO2 nanocomposites. Polymer.

[20] Dorigato A., Pegoretti A., Kolařík J. (2010). Nonlinear tensile creep of linear low density polyethylene/fumed silica nanocomposites: Time-strain superposition and creep prediction. Polym. Compos..

[21] Dorigato A., Pegoretti A., Fambri L., Slouf M., Kolarik J. (2010). Cycloolefin copolymer/fumed silica nanocomposites. J. Appl. Polym. Sci..

[22] Dorigato A., D’Amato M., Pegoretti A. (2012). Thermo-mechanical properties of high-density polyethylene—Fumed silica nanocomposites: Effect of filler surface area and treatment. J. Polym. Res..

[23] Benabid F.Z., Kharchi N., Zouai F., Mourad A.H.I., Benachour D. (2019). Impact of co-mixing technique and surface modification of ZnO nanoparticles using stearic acid on their dispersion into HDPE to produce HDPE/ZnO nanocomposites. Polym. Polym. Compos..

[24] Guo G., Finkenstadt V.L., Nimmagadda Y. (2019). Mechanical properties and water absorption behaviour of injection-molded wood fiber/carbon fiber high-density polyethylene hybrid composites. Adv. Compos. Hybrid Mater..

[25] Huang R., Xu X., Lee S., Zhang Y., Kim B.J., Wu Q. (2013). High density polyethylene composites reinforced with hybrid inorganic fillers: Morphology, mechanical and thermal expansion performance. Materials.

[26] Kolařík J., Kruliš Z., Šlouf M., Fambri L. (2005). High-density polyethylene/cycloolefin copolymer blends. Part 1: Phase structure, dynamic mechanical, tensile, and impact properties. Polym. Eng. Sci..

[27] Fenni S.E., Caputo M.R., Müller A.J., Cavallo D. (2022). Surface Roughness Enhances Self-Nucleation of High-Density Polyethylene Droplets Dispersed within Immiscible Blends. Macromolecules.

[28] Jancar J., Fekete E., Hornsby P.R., Jancar J., Pukánszky B., Rothon R.N. (1999). Mineral Fillers in Thermoplastics I: Raw Materials and Processing.

[29] Mejia E., Cherupurakal N., Mourad A.-I., al Hassanieh S., Rabia M. (2021). Effect of Processing Techniques on the Microstructure and Mechanical Performance of High-Density Polyethylene. Polymers.

[30] Mozumder M.S., Mourad A.-I., Mairpady A., Pervez H., Haque M.E. (2018). Effect of TiO_2_ Nanofiller Concentration on the Mechanical, Thermal and Biological Properties of HDPE/TiO_2_ Nanocomposites. J. Mater. Eng. Perform..

[31] Pervez H., Mozumder M.S., Mourad A.-I. (2016). Optimization of injection molding parameters for HDPE/TiO_2_ nanocomposites fabrication with multiple performance characteristics using the Taguchi method and grey relational analysis. Materials.

[32] Mourad A.-I., Mozumder M.S., Mairpady A., Pervez H., Kannuri U.M. (2017). On the Injection Molding Processing Parameters of HDPE-TiO_2_ Nanocomposites. Materials.

[33] Ansys Edupack Data Basem High Density Polyethylene/HDPE/PE-HD (Homopolymer, General Purpose, Molding Extrusion). https://www.sciencedirect.com/topics/engineering/high-density-poly-ethylene.

[34] Taşdemir M., Ersoy S. (2015). Mechanical, morphological and thermal properties of HDPE polymer composites filled with talc, calcium carbonate and glass spheres. Rom. J. Mater..

[35] Mehrjerdi A.K., Adl-Zarrabi B., Cho S., Skrifvars M. (2013). Mechanical and thermo-physical properties of high-density polyethylene modified with talc. J. Appl. Polym. Sci..

[36] Awan M., Shakoor A., Rehan M.S., Gill Y.Q. (2021). Development of HDPE composites with improved mechanical properties using calcium carbonate and NanoClay. Phys. B Condens. Matter.

[37] Leong Y.W., Abu Bakar M.B., Ishak Z.M., Ariffin A., Pukanszky B. (2004). Comparison of the mechanical properties and interfacial interactions between talc, kaolin, and calcium carbonate filled polypropylene composites. J. Appl. Polym. Sci..

